# Supplementary material for: Charge neutralization of the active site glutamates does not limit substrate binding and transport by small multidrug resistance transporter EmrE
Source: J Biol Chem. 2022 Dec 16;299(2):102805. doi: 10.1016/j.jbc.2022.102805 (PMC9860125; doi:10.1016/j.jbc.2022.102805)
Supplement: Supporting information [file mmc1.docx]

**Supporting Information:**

Raw NMR data can be found on the BMRbig database under the accession number, [bmrbig70](https://bmrbig.bmrb.io/released/bmrbig70).

Online datasets can be found on MendeleyData at: <https://doi.org/10.17632/gs238vhsx4.1>

Until the work is published, use this link: <https://data.mendeley.com/datasets/gs238vhsx4/draft?a=40f2fb3a-3bae-4755-b16b-2f285f665666>


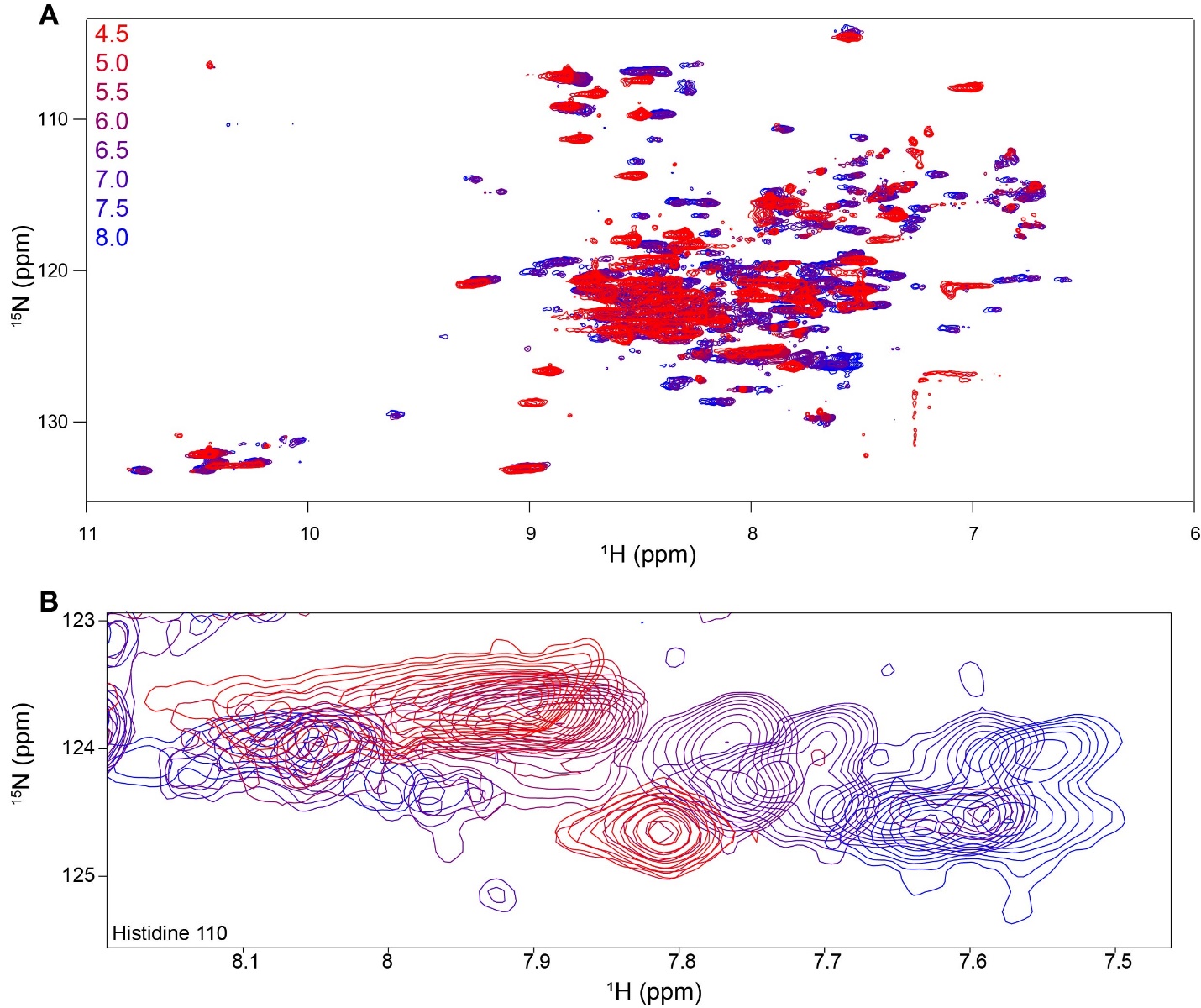


**Figure S1: pH titration of propidium-bound WT-EmrE.** A) ^1^H^15^N-TROSY HSQC spectra of PP^2+^-bound WT-EmrE in q=0.33 DMPC/DHPC isotropic bicelles were recorded on a Varian 800 MHz spectrometer at 45°C from low to high pH (4.5-8.0, colors as shown). These spectra exhibit chemical shift changes indicative of protonation in addition to the already-bound PP^2+^. B) The shifts of H110 reveal that EmrE titrates throughout the pH range in this experiment and the lack of signal from residues, such as A10, near neutral pH are the result of line broadening from chemical exchange and not loss of protein.


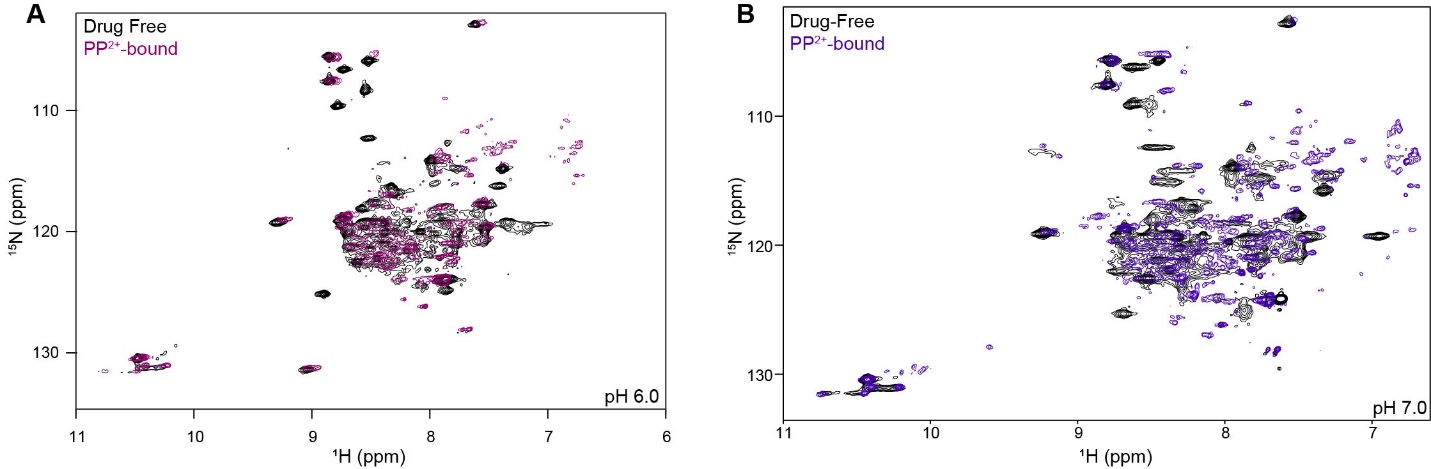


**Figure S2: Faster dynamics at physiological pH with propidium-bound WT-EmrE.** At pH 6.0 (A) and pH 7.0 (B), propidium-bound WT-EmrE has slow-intermediate timescale dynamics resulting in increased line broadening. The dynamics of the planar ligand within the transport pore combined with the exchange between different protonation states near the pKa likely contribute to this phenomenon.


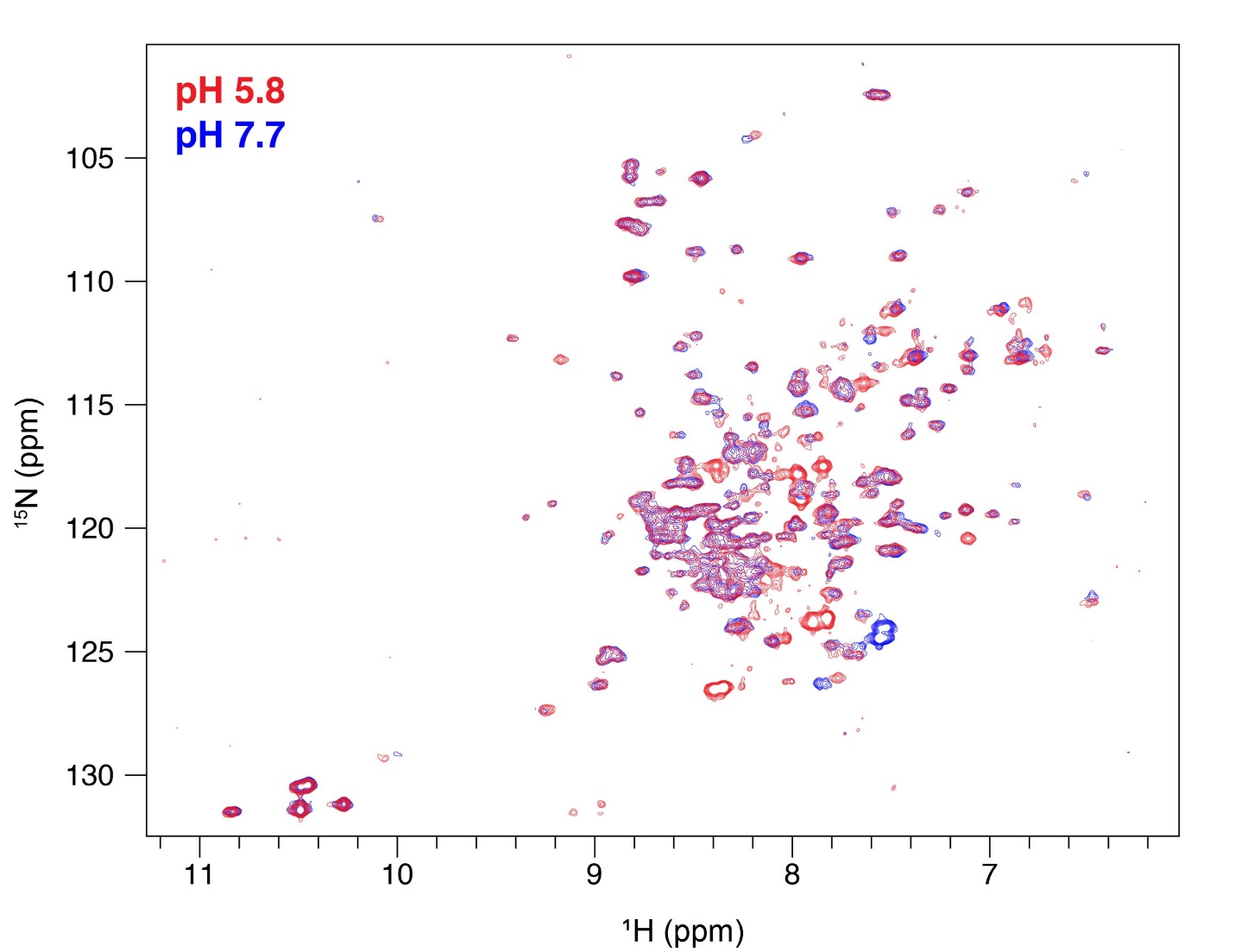


**Figure S3: E14Q-EmrE at low and high pH shows C-terminal tail titration.** E14Q-EmrE removes the effect of glutamate protonation in the active site in EmrE. Chemical shift differences between low and high pH are limited to residues in the C-terminal tail, consistent with the pKa of the C-terminal residue H110.


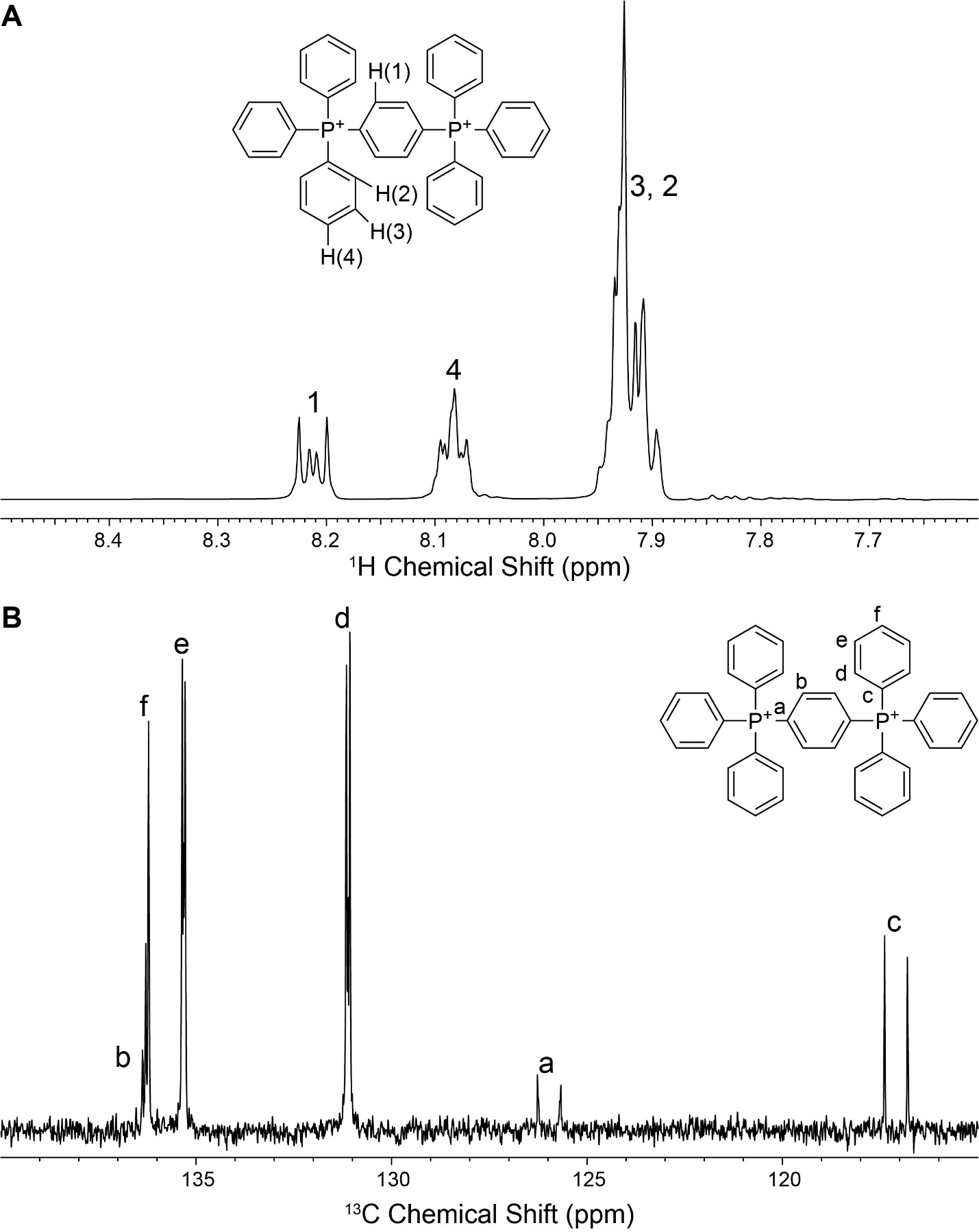


**Figure S4: 1D NMR dTPP^2+^.** ^1^H (A) and ^13^C (B) 1D NMR spectra of dTPP^2+^ confirm the symmetry of the molecule. Spectra were obtained on a Bruker Avance III 600MHz spectrometer at 25°C.


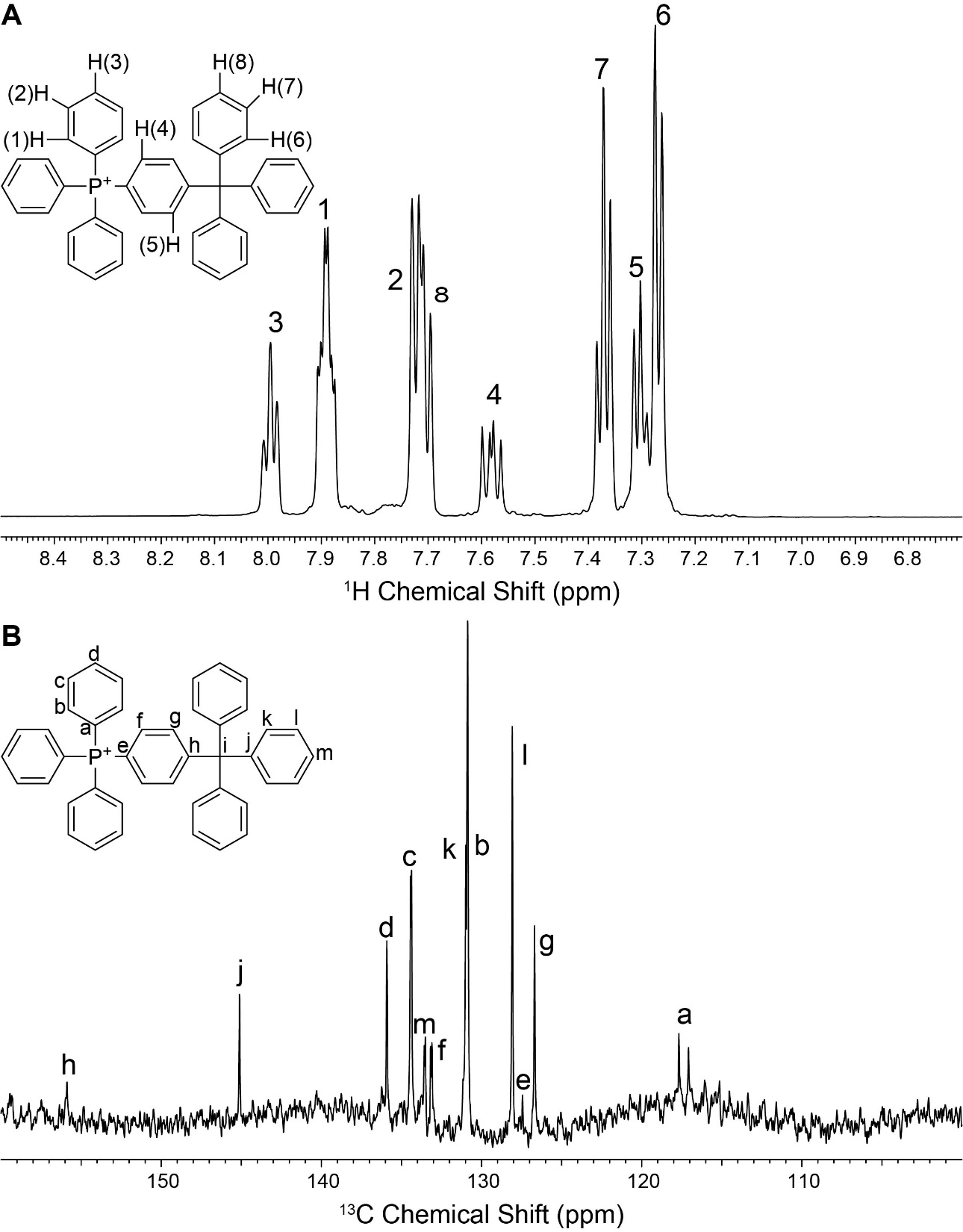


**Figure S5: 1D NMR of dTPP^+^.** ^1^H (A) and ^13^C (B) 1D NMR spectra of dTPP^+^ confirm the symmetry of the molecule. Spectra were obtained on a Bruker Avance III 600MHz spectrometer at 25°C.
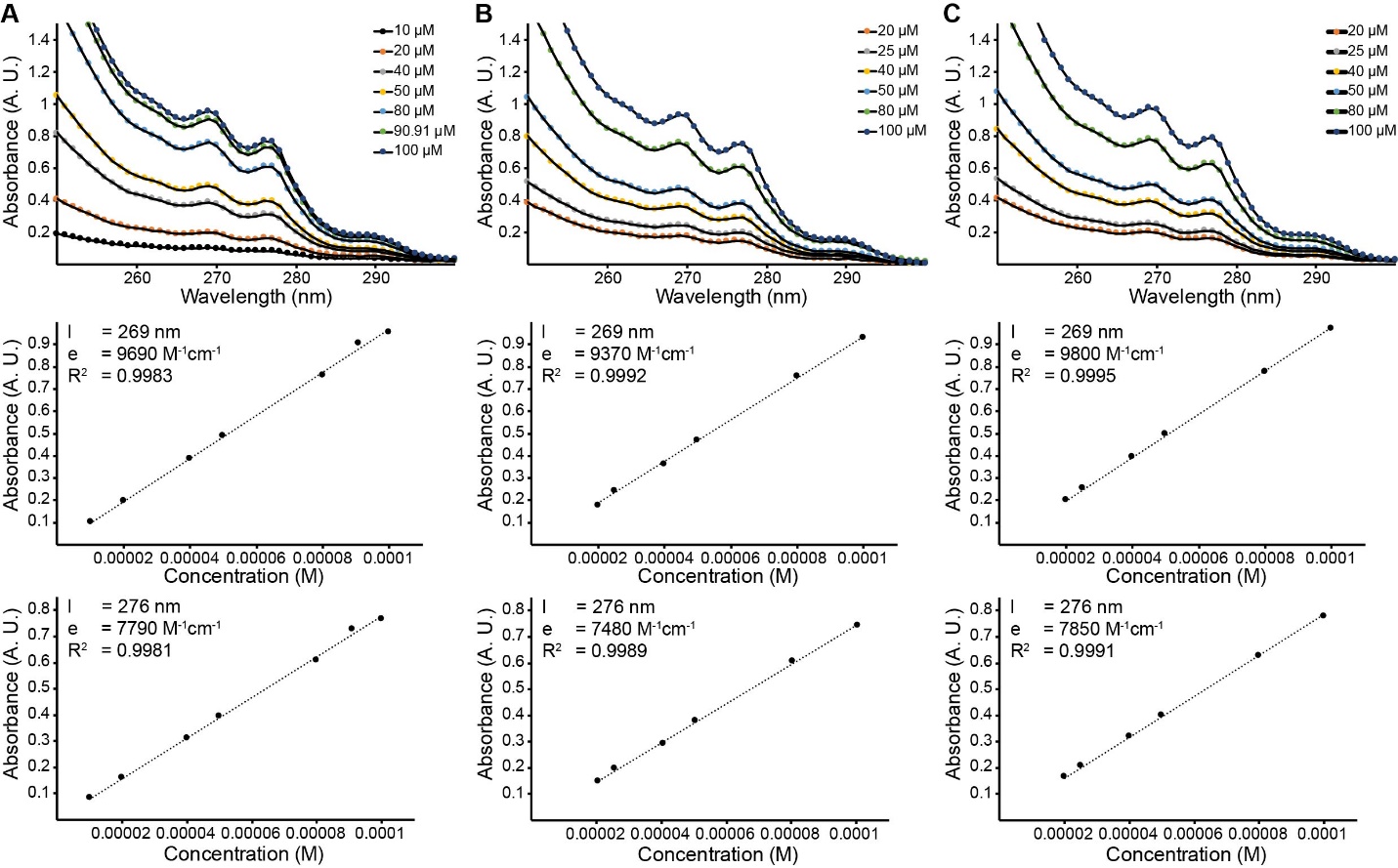


**Figure S6: Extinction coefficient trials of dTPP^2+^.** Trials 1-3 (A-C, respectively) of extinction coefficient experiments for dTPP^2+^. A summary of the values from this analysis can be found in Table S1.


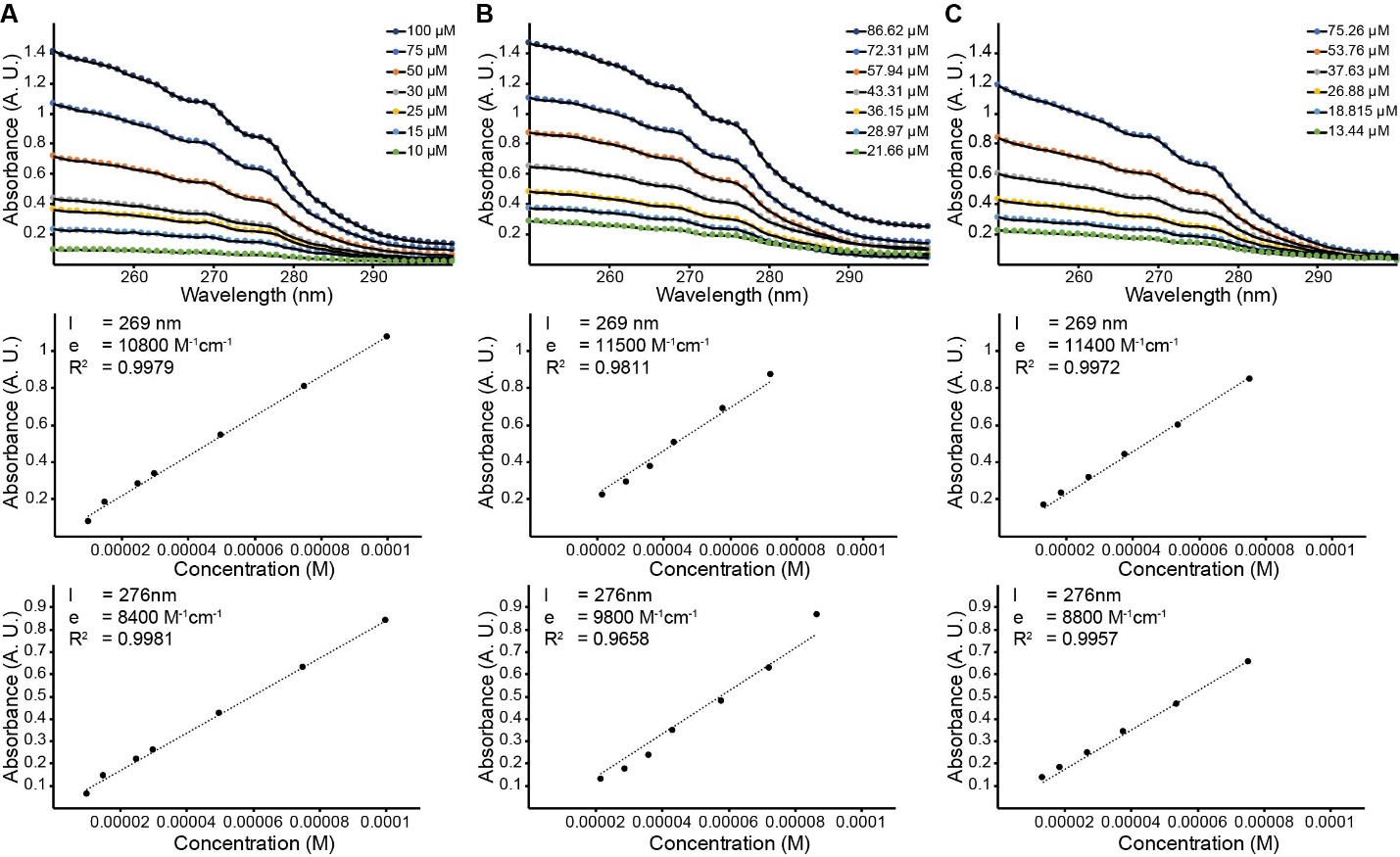


**Figure S7: Extinction coefficient trials of dTPP^+^.** Trials 1-3 (A-C, respectively) of extinction coefficient experiments for dTPP^+^. A summary of the values from this analysis can be found in Table S1.


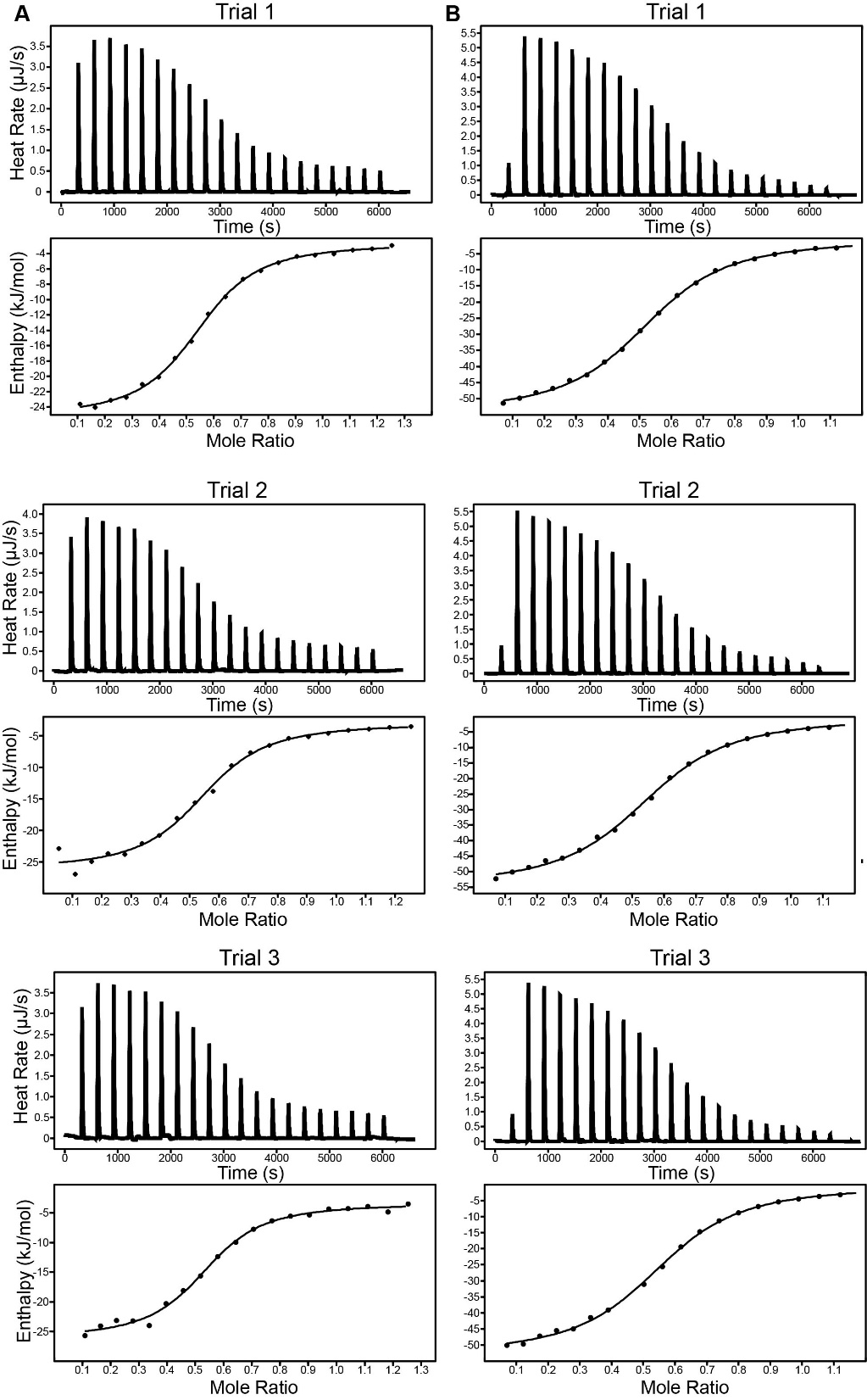


**Figure S8: ITC trials of dTPP derivatives.** ITC curves from Trials 1-3 of dTPP^2+^ (A) and dTPP^+^ (B) at pH 7.0. Trial 1 (top) is repeated from Fig. 4 of the main text to facilitate direct comparison of all replicates.


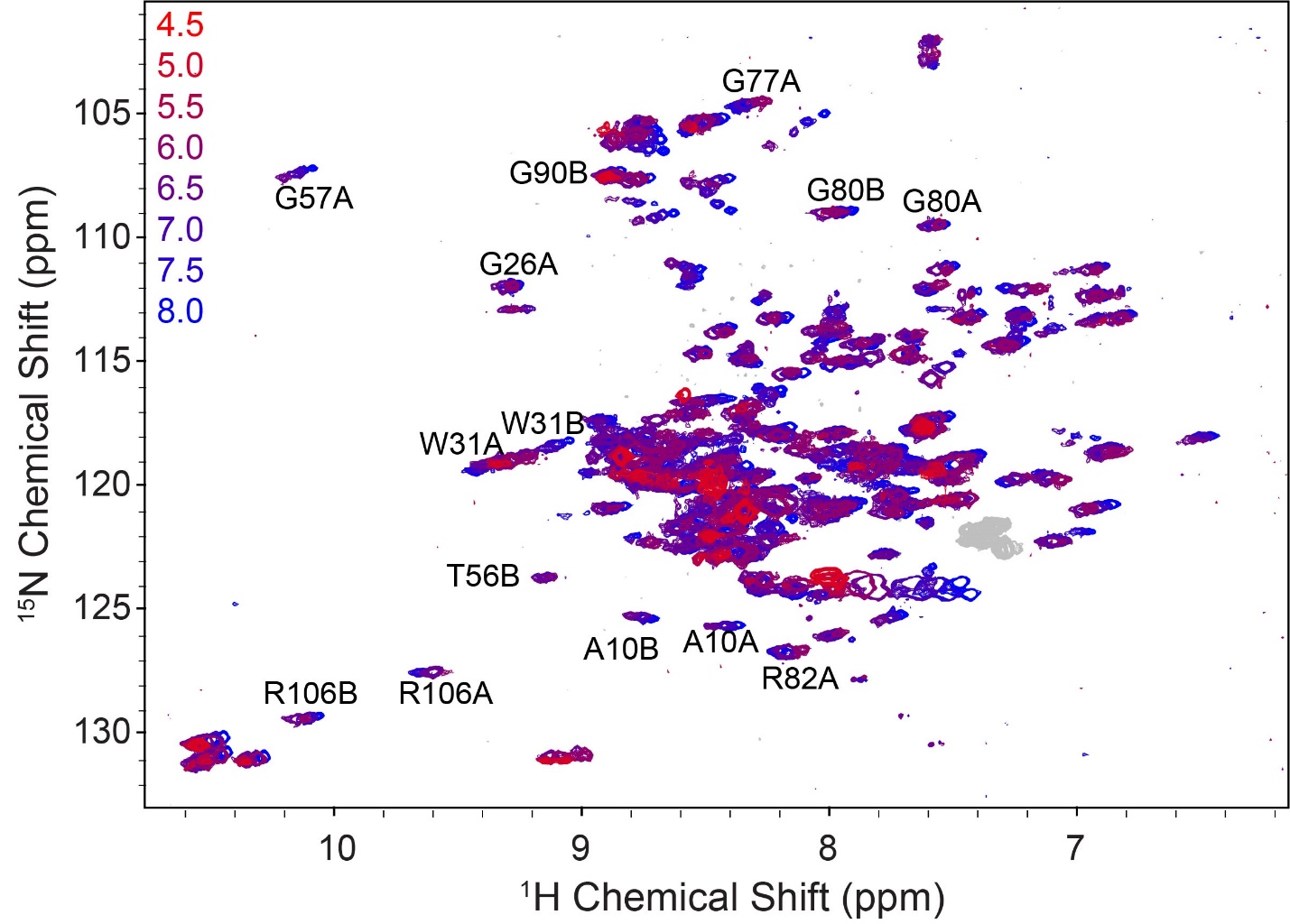


**Figure S9: pH titration of dTPP^+^-bound WT-EmrE.** ^1^H^15^N-TROSY HSQC spectra of dTPP^+^-bound WT-EmrE in q=0.33 DMPC/DHPC isotropic bicelles were recorded on a Varian 800 MHz spectrometer at 45°C from low to high pH (4.5-8.0, colors as shown). These spectra exhibit chemical shift changes indicative of protonation in addition to the already bound dTPP^+^. Peaks were assigned using original assignments from TPP^+^-bound WT-EmrE pH titrations.


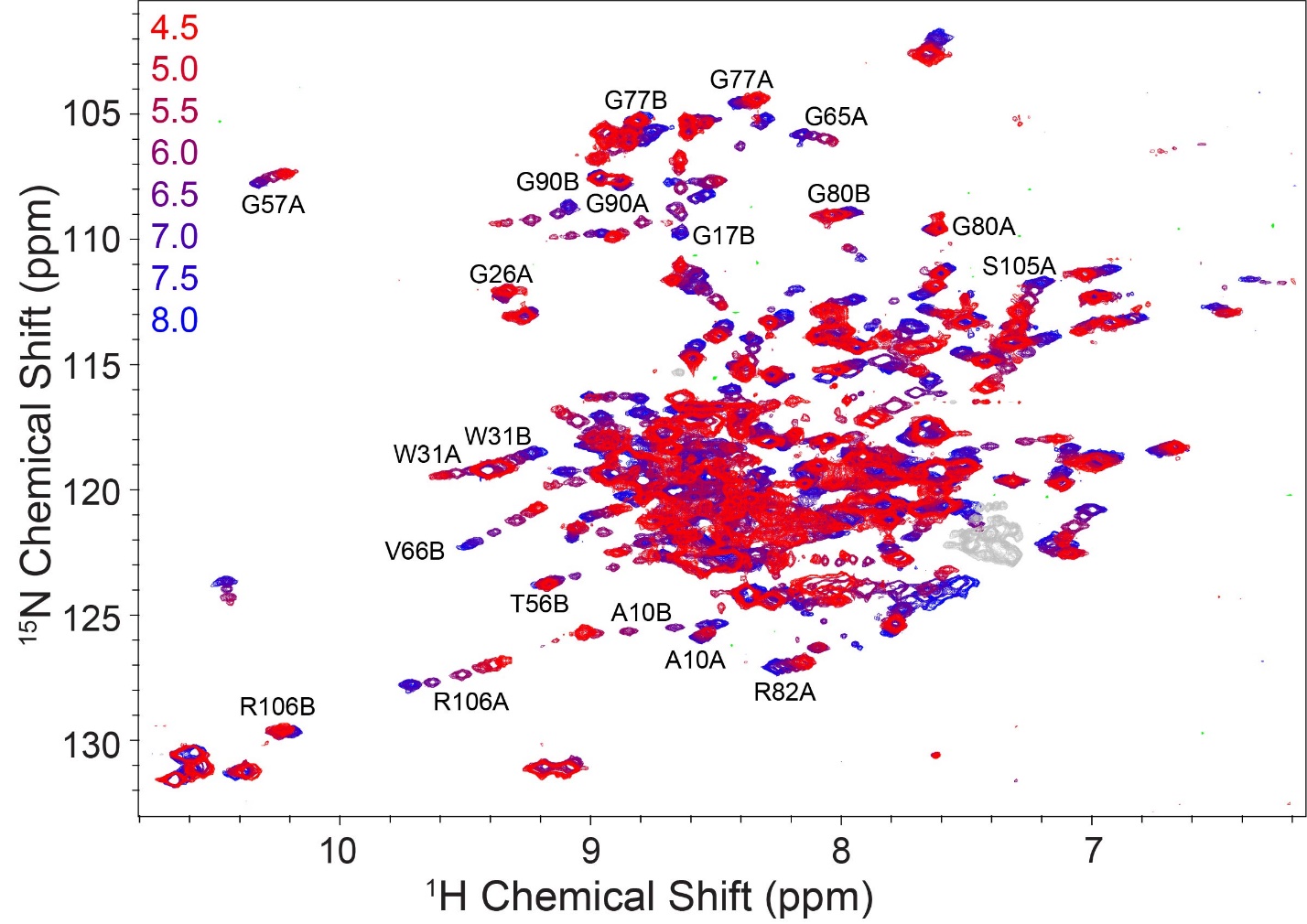


**Figure S10: pH titration of dTPP^2+^-bound WT-EmrE.** ^1^H^15^N-TROSY HSQC spectra of dTPP^2+^-bound WT-EmrE in q=0.33 DMPC/DHPC isotropic bicelles were recorded on a Varian 800 MHz spectrometer at 45°C from low to high pH (4.5-8.0, colors as shown). These spectra exhibit chemical shift changes indicative of protonation in addition to the already bound dTPP^2+^. Peaks were assigned using original assignments from TPP^+^-bound WT-EmrE pH titrations.


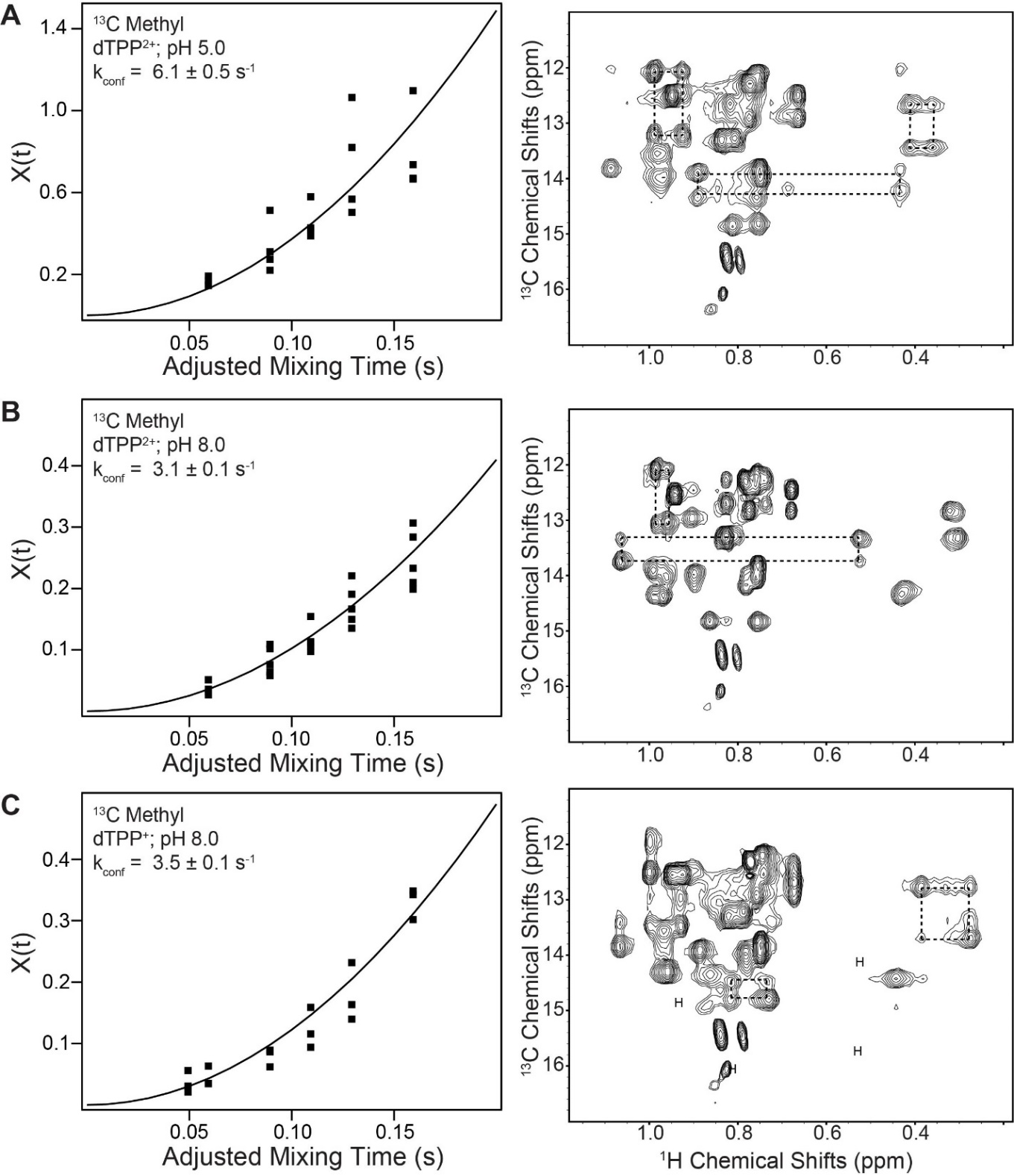


**Figure S11: Methyl ZZ-exchange spectroscopy of dTPP derivatives at low and high pH.** The rate of alternating access of ILV-labeled WT-EmrE saturated with either dTPP^2+^ (pH 5.0, A; pH 8.0, B) or dTPP^+^ (pH 8.0, C) were analyzed using methyl ZZ-exchange spectroscopy. Sample spectral planes with intermediate mixing times are shown on the right and boxes are drawn to highlight methyl resonances for which auto peaks for state A and state B and the cross peaks are all sufficiently resolved to use in quantitative analysis. The auto and cross peak ratio, calculated as described in the methods, is shown as a function of mixing time on the left along with the best fit.


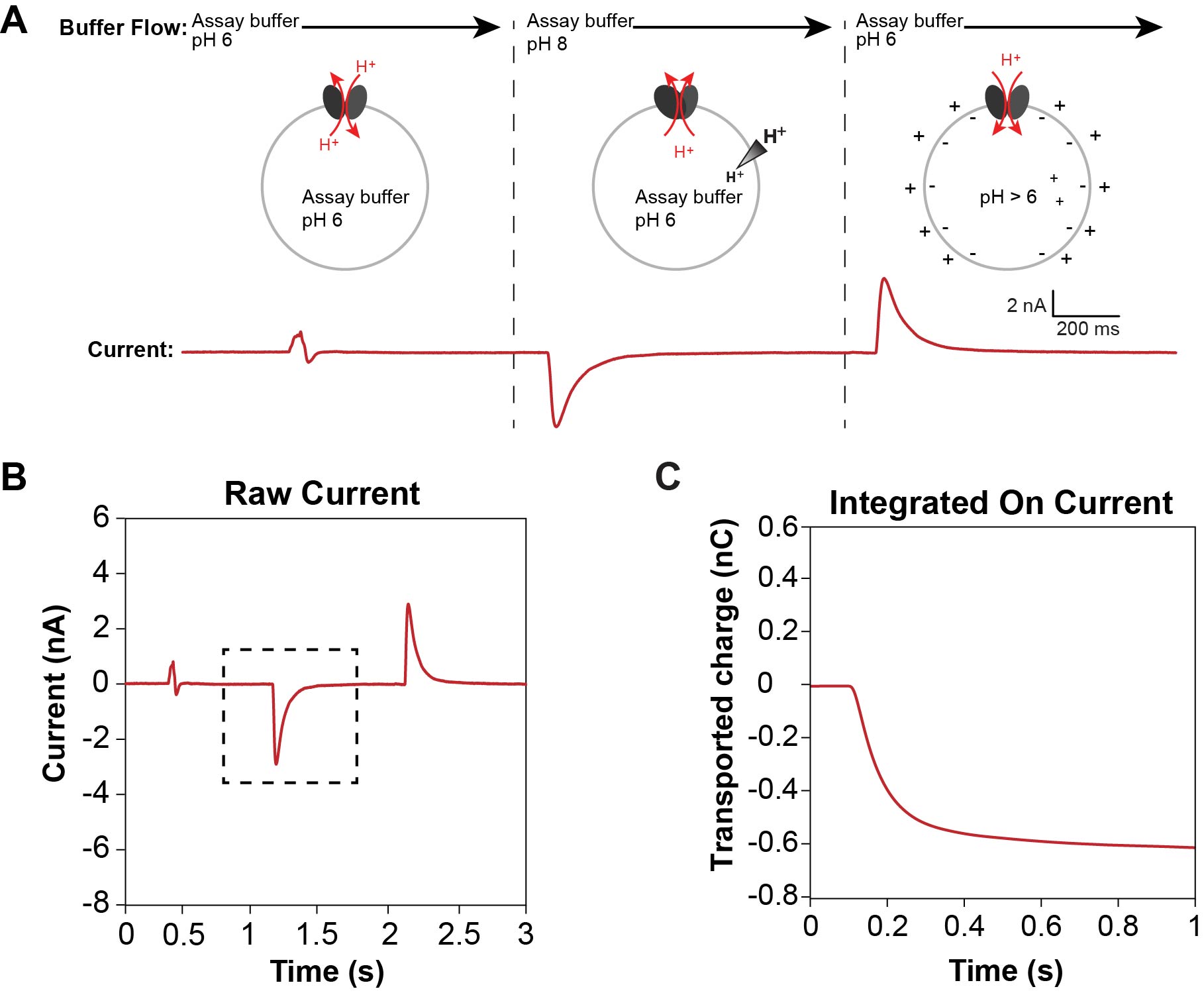


**Figure S12: SSME Experimental Scheme.** Solid-supported membrane electrophysiology experiments utilize buffer perfusions over liposomes adhered to a membrane coating a gold electrode. Perfusions occur in three stages with the current recorded throughout. First, the buffer into which the liposomes have been equilibrated is run over the outside of the liposomes resulting in minimal signal. Second, a buffer used to establish the desired gradient condition is perfused and the resulting signal generates the “on current” (A, middle; B, box), which is the signal due to transport driven by that gradient condition. Finally, the initial buffer is again perfused over the outside to alleviate any built-up charge in the liposome due to transport. The transported charge is then calculated from the integrated on current (C).


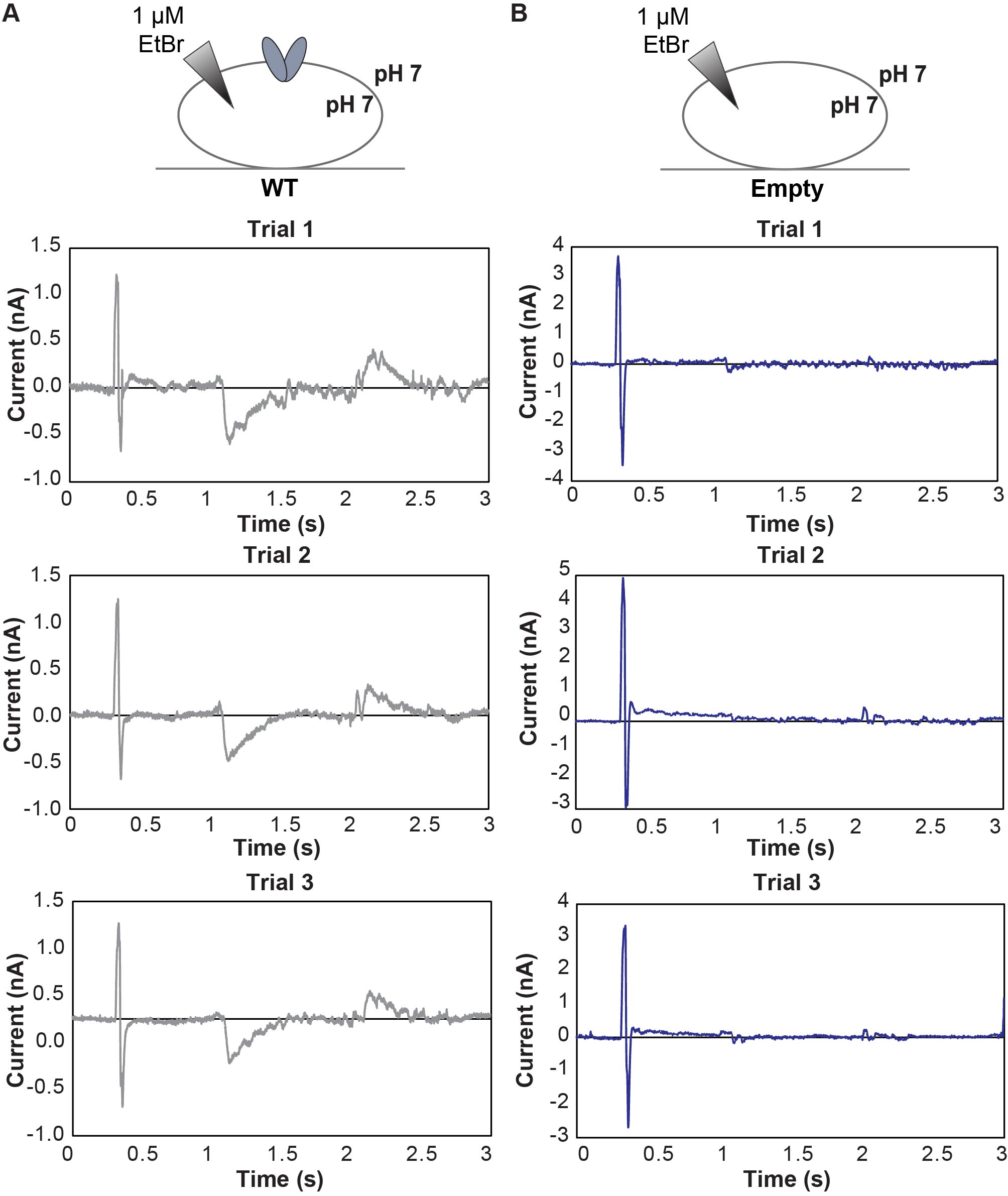


**Figure S13: SSME trials of Eth^+^ transport.** Trials of 1 µM Ethidium transport at pH 7 using three separately prepared sensors each of WT-EmrE proteoliposomes (A) and empty liposomes (B). Transport currents for the WT sensors overlay well demonstrating reproducibility, while Empty sensors show minimal signal confirming that minimal interactions are taking place with the lipid membrane at this concentration.


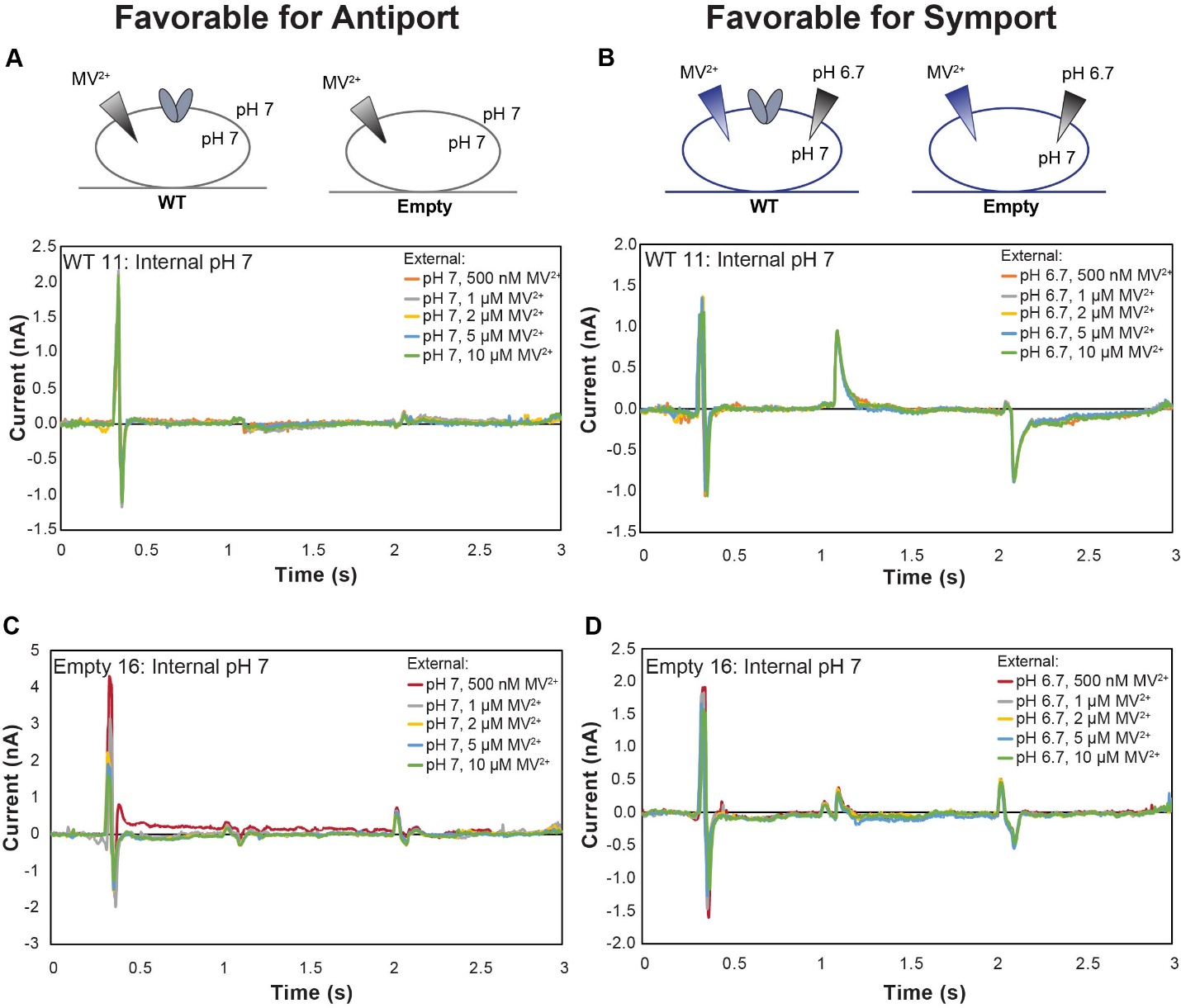


**Figure S14: SSME transport of the +2 MV^2+^.** When conditions favorable for driving antiport are applied with the +2 substrate MV^2+^, minimal signal is detected as expected for net neutral antiport of two protons out of the liposome for each MV^2+^ molecule that enters the liposome (A). The same sensor was perfused with buffers with the same MV^2+^ concentrations but pH 6.7 to create 2-fold proton gradients in the same direction as the drug gradient. This drives symport (B). A sensor containing empty liposomes was perfused with the same buffering conditions to confirm that the signal observed is due to EmrE-mediated transport (C, D). Note that MV^2+^ is only added on one side of the liposome, creating a very large inward drug gradient in all experiments.


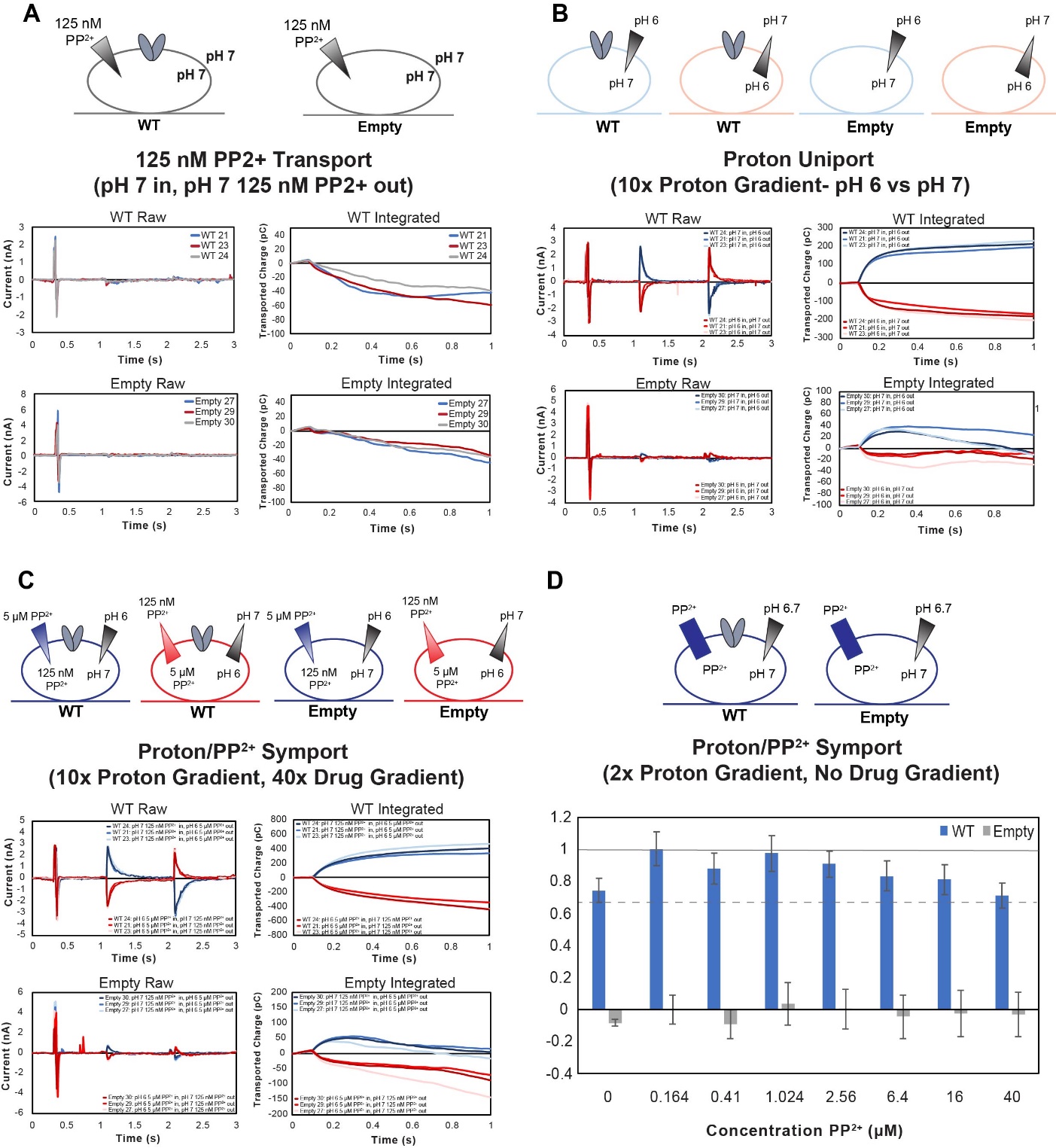


**Figure S15: SSME transport of the +2 PP^2+^.** A-C) Overlays of the different sensors averaged for replicates of PP^2+^ antiport (A, transport experiment set up as in Fig. 7A with no pH gradient and PP^2+^ added externally), proton uniport (B), and proton/PP^2+^ symport (C) show a high degree of reproducibility for the assay. Note the different axis scale in each panel, where PP^2+^/2H^+^ antiport leads to very little signal on the same scale as empty liposomes under the transport conditions (A), whereas proton uniport (B) or proton/PP^2+^ symport (C) show much larger signal that is well above the signal recorded for empty liposomes. D) Normalized transported charge for proton/PP^2+^ symport under a lesser proton gradient (2x) show the increased transport in the presence of PP^2+^ but with smaller magnitude, consistent with the reduced driving force. In contrast, the total net transport is not significantly affected by substrate (PP^2+^) concentration (D) if the driving force (2-fold proton gradient) remains constant.

**Table S1: Extinction coefficient trials**

|  | **dTPP^2+^ ε_269_**  **(M^-1^cm^-1^)** | **dTPP^2+^ ε_276_**  **(M^-1^cm^-1^)** | **dTPP^+^ ε_269_**  **(M^-1^cm^-1^)** | **dTPP^+^ ε_276_**  **(M^-1^cm^-1^)** |
| --- | --- | --- | --- | --- |
| Trial 1 | 9692 | 7793 | 10778 | 8434 |
| Trial 2 | 9366 | 7481 | 12188 | 9672 |
| Trial 3 | 9796 | 7852 | 11361 | 8804 |
| Average | 9618 | 7708 | 11442 | 8970 |
| Standard Deviation | 224 | 199 | 709 | 635 |
| Consensus Value | 9600 ± 200 | 7700 ± 200 | 11400 ± 700 | 9000 ± 600 |

**Table S2: ITC trial summary**

|  | **K_D_** | ***n*** | **∆G** | **∆H** | **∆S** | **[EmrE]_monomer_** | **[Substrate]** | ***c*-value** |
| --- | --- | --- | --- | --- | --- | --- | --- | --- |
|  | **µM** |  | **kJ/mol** | **kJ/mol** | **J/mol•K** | **mM** | **mM** |  |
| dTPP^2+^ Trial 1 | 8.38 ± 1.38 | 0.54 ± 0.01 | -30.9 ± 0.4 | -22.5 ± 0.7 | 26.42 | 0.38 | 1.36 | 25 ± 4 |
| dTPP^2+^ Trial 2 | 11.00 ± 3.55 | 0.54 ± 0.02 | -30.2 ± 0.9 | -24.8 ± 1.7 | 16.97 | 0.38 | 1.36 | 19 ± 6 |
| dTPP^2+^ Trial 3 | 7.59 ± 2.91 | 0.53 ± 0.02 | -31.2 ± 1.0 | -22.7 ± 1.6 | 26.75 | 0.38 | 1.36 | 27 ± 10 |
| dTPP^+^ Trial 1 | 9.14 ± 1.03 | 0.53 ± 0.01 | -30.7 ± 0.3 | -53.8 ± 1.3 | -72.5 | 0.29 | 0.96 | 17 ± 2 |
| dTPP^+^ Trial 2 | 9.47 ± 1.87 | 0.54 ± 0.01 | -30.6 ± 0.5 | -54.2 ± 2.4 | -74.2 | 0.29 | 0.96 | 16 ± 3 |
| dTPP^+^ Trial 3 | 8.99 ± 1.57 | 0.56 ± 0.01 | -30.7 ± 0.5 | -52.4 ± 2.0 | -68.15 | 0.29 | 0.96 | 18 ± 3 |
| ^2^H-dTPP^2+^ Trial 1 | 13.42 ± 2.58 | 0.52 ± 0.01 | -29.7 ± 0.5 | -27.8 ± 1.3 | 6.02 | 0.40 | 1.20 | 15 ± 3 |
| ^2^H-dTPP^2+^ Trial 2 | 12.48 ± 2.04 | 0.51 ± 0.01 | -29.9 ± 0.4 | -27.6 ± 1.0 | 7.08 | 0.40 | 1.20 | 16 ± 3 |
| ^2^H-dTPP^2+^ Trial 3 | 12.00 ± 1.57 | 0.51 ± 0.01 | -30. 0 ± 0.3 | -27.7 ± 0.8 | 7.25 | 0.40 | 1.20 | 17 ± 2 |
